# Case Report Highlighting Cardiovascular Effects of Concomitant Use of Methamphetamine and Marijuana

**DOI:** 10.7759/cureus.27866

**Published:** 2022-08-10

**Authors:** Sophia Fontana, Ladan Panahi, George Udeani, Salim Surani, Deepa Desai

**Affiliations:** 1 Pharmacy Practice, Texas A&M University, Kingsville, USA; 2 Medicine, Texas A&M University, College Station, USA; 3 Internal Medicine, Pulmonary Associates, Corpus Christi, USA; 4 Pharmacy, Ascension Health, Round Rock, USA

**Keywords:** tachycardia, congestive heart failure, chf, cardiotoxicity, methamphetamine, cannabis, marijuana

## Abstract

We present the case of a 51-year-old male admitted for cardiovascular complications in the face of concomitant chronic methamphetamine and cannabis use. Upon further assessment, the patient exhibited cardiotoxicity, including acute to chronic congestive heart failure (CHF) exacerbation, hypercoagulable state, and electrolyte abnormalities. Cardiotoxicity secondary to chronic methamphetamine use has been established. However, marijuana's cardiovascular effects have not been well established. Even less information exists about the simultaneous use of methamphetamine and cannabis. With increasing interest in the use of marijuana for medical purposes, it is imperative to study any corresponding toxicity and adverse effect profile. The worldwide pattern of drug co-administration also brings the importance of this topic to light. This case report serves to provide insight into this information gap.

## Introduction

In the United States, during 2015-2018, approximately 1.6 million adults, on average, used methamphetamine each year [1]. Among adults using methamphetamine within the past year, the prevalence of use or misuse of other substances, including cannabis use, was 68.7% [1]. In 2011, about one-fifth of methamphetamine-related visits involved combinations with marijuana [2]. The trend of drug coadministration is increasing worldwide [3]. A higher likelihood of methamphetamine abuse is associated with existing marijuana abuse, with an odds ratio of 3.33 [4].

In addition, a 12‐fold increase in the number of annual hospitalizations due to heart failure (HF) secondary to methamphetamine use from 2002 to 2014 (547 to 6625, +1111%; P<0.0001) was observed [5]. The prevalence of heart failure due to methamphetamine increased from 2.5 cases per 1000 in 2010 to 7.4 cases per 1000 in 2014 (P<0.0001) [5]. Thomas et al. found that in patients using marijuana and methamphetamine concomitantly, there was approximately a 20-fold increase in the diagnosis of methamphetamine-associated heart failure [5].

There are mixed findings in the literature when evaluating the cardiovascular effects of cannabis use. A 9.5% association of adverse effects due to cardiovascular disorders was reported by a multicenter study published in 2011 in cannabis-related hospital admissions [6]. The U.S. National Vital Statistics for 1990-2014 data demonstrated that mortality rates in states where marijuana is legal showed a 2.3% and 1.3% increase in cardiac mortality among men and women, respectively, compared to states where marijuana is not legal [6]. The association between the development of heart failure or exacerbation and marijuana use has not been fully demonstrated [6]. However, marijuana use was identified as an independent predictor of heart failure with an odds ratio of 1.1 (95% confidence interval, 1.03-1.18, p-value < 0.01) in a retrospective database review of patients diagnosed with heart failure at discharge [6].

Even less information exists about the simultaneous use of methamphetamine and marijuana. With increasing interest in the use of marijuana for medical purposes and the worldwide pattern of drug coadministration, it is imperative to study any corresponding toxicity and adverse effects from the concomitant use of methamphetamines and marijuana.

Methamphetamine

Methamphetamine is a potent stimulant that is also a synthetic amine [7]. Amphetamine metabolites are active but are unlikely to cause additional effects as they peak at concentrations substantially lower than the ingested drug [8]. The methyl moiety that differentiates methamphetamine from amphetamine analogs also increases its penetration across the blood-brain barrier [9]. Initial reports of amphetamine-type stimulants (ATSs) use date back before World War II [10]. The Comprehensive Drug Abuse Prevention and Control Act of 1970 aimed to restrict the legal and medical uses of prescribed amphetamines [10]. Methamphetamine ice made its way into Hawaii through imports from the Philippines and Southeast Asia during the 1980s [10]. Currently, methamphetamine is the stimulant with the highest illegal manufacture, distribution, and abuse rate in America [7]. Methamphetamine occurs in two isomeric forms: d-methamphetamine and l-methamphetamine [11]. These two isomeric forms occur due to the chiral center of methamphetamine [11]. The form that is manufactured for illicit use and is more potent is the d-isomer [12]. While methamphetamine is often referred to as "speed," "crystal," "crank," and "go," the terms "ice" or "crystal meth" are used in reference to d-methamphetamine, which appears as translucent crystals [7,8]. While methamphetamine is commonly taken orally or injected, the d-methamphetamine form is more widely used as vapor inhalation [8].

Marijuana

For over 6,000 years, the cannabis plant has been cultivated by humans [13]. While the main active compound in marijuana is delta-9-tetrahydrocannabinol (d-9-THC), the cannabis plant contains 60 cannabinoid compounds out of over 400 different compounds identified [13]. Although medical interest in marijuana has increased over the years, the chemical structure was first identified in 1965, and during the 19th century, it appeared in Western medicine [14]. The discovery of the endogenous cannabinoid system and cannabinoid system in the 1990s added to our understanding of marijuana [14]. The d-9-THC compound is active on two primary cannabinoid receptors: cannabinoid receptor type 1 (CB1R) and cannabinoid receptor type 2 (CB2R) [13]. CB1R is found in the brain and peripheral tissues, some of which include cardiac, hepatic tissue, gastrointestinal, and vascular endothelium [15]. The CB2Rs are found primarily in immune cells [15]. THC is found to have acted as a partial agonist on CB1R and CB2R. Endocannabinoids typically modulate the release of neurotransmitters at CB1R and CB2R [13]. In the brain, THC may inhibit GABA, increasing dopamine, glutamate, and acetylcholine [13].

## Case presentation

Introduction to patient case

A 51-year-old male was seen in the emergency department with an acute chronic congestive heart failure (CHF) exacerbation characterized by lower extremity pain and swelling. The patient reported chronic bilateral lower extremity swelling that worsened, prompting him to report to the emergency department. The patient was alert and cooperative and stated that he had been compliant with his furosemide (40 mg by mouth every morning).

**Figure 1:**
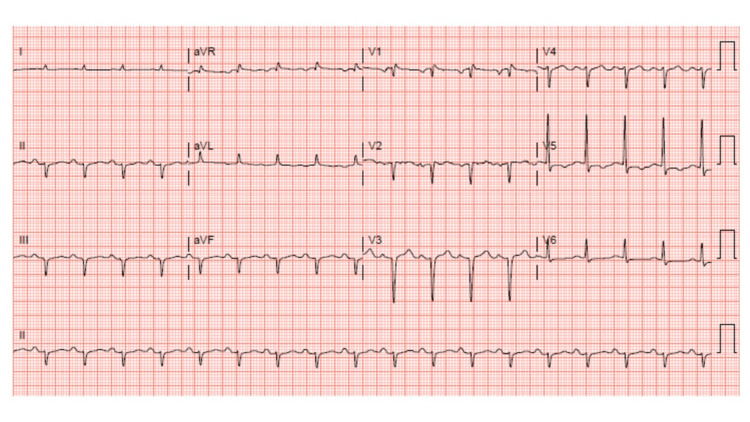
Electrocardiogram at hospital admission

The patient was initially hypotensive with a blood pressure of 91/71 mmHg, tachycardic with a heart rate of 111 beats per minute, afebrile at 97.8 °F, and a respiratory rate of 16 breaths per minute with an initial oxygen saturation of 91% on room air. Saturation rose to 94% on a 4-L nasal cannula and eventually to 100% on room air by day 4 of hospital admission. The findings of the electrocardiogram (ECG), Figure 1, performed at admission, were sinus tachycardia and non-specific T-wave abnormalities.

The presentation was notable for orthopnea, pitting edema in the lower extremities, and generalized weakness. Past medical history included pulmonary embolism (PE), deep vein thrombosis (DVT), alcoholic cirrhosis, loculated pneumothorax, pleural effusion, ascites, and chronic CHF. The significant social history reported by the patient includes chronic alcohol, methamphetamine, and marijuana abuse, as well as being homeless. The patient stated he quit alcohol use 60 days prior but used to be a heavy alcohol user. The patient has reported heavy drinking since 2020, and prior to that, he was only a social drinker. The patient stated they were drinking Everclear mixed drinks, approximately 4 to 6 liters per week. Blood toxicology was negative for acetaminophen, salicylates, and alcohol. A urine toxicology test was performed for amphetamine, but no methamphetamine urine screen was performed. The urine toxicology result showed no amphetamine present, as noted in Table 1. The patient also reports occasional marijuana use, and the urine drug screen was positive for cannabinoids 20 days earlier and on the day of admission. Family history was significant for CHF, although the patient could not specify details regarding family history. The patient reports taking scheduled furosemide for edema, midodrine as needed for hypotension, and scheduled apixaban for a recent PE diagnosis. It was assessed that the patient had not been compliant with apixaban, per patient report, which had been initiated during their prior admission to a different hospital 60 days prior for the treatment of a bilateral PE. An echocardiogram performed 20 days before hospital admission reported a left ventricular ejection fraction (LVEF) of 10-15% with grade II diastolic dysfunction. An echocardiogram was not performed during the visit as the patient was discharged against medical advice.

**Table 1:** Serum and urine toxicology screening The urine and serum toxicology screenings were performed on the day of admission.

Test performed	Result
Serum toxicology	
Acetaminophen level (mcg/mL)	<1
Salicylate level (<20 mg/dL)	10.9
Alcohol level (<10 mg/dL)	<10
Tricyclic qualitative (<300 ng/mL)	Negative
Urine toxicology screen	
Amphetamine (1000 ng/mL)	Negative
Barbiturate (<200 ng/mL)	Negative
Benzodiazepine (<200 ng/mL)	Negative
Cannabinoid (<50 ng/mL)	Presumptive positive
Cocaine (<300 ng/mL)	Negative
Methadone (<300 ng/mL)	Negative
Opiate (<300 ng/mL)	Negative
Phencyclidine (<25 ng/mL)	Negative

The patient is assessed to have a complex list of medical problems, including acute CHF exacerbation, left lower extremity cellulitis with possible secondary sepsis, history of PE, acute kidney injury (AKI), lactic acidosis, hyponatremia, hypochloremia, normocytic anemia, hypocalcemia, hypokalemia, elevated alkaline phosphatase, elevated total bilirubin, supratherapeutic INR, substance abuse, and chronic hypotension. Pertinent lab values are depicted in Table 2. Lower extremity swelling was suspected to be secondary to CHF and potentially cellulitis. CHF was classified as ACC/AHA stage D and NYHA Class IV 60 days prior. AKI was suspected of having occurred secondary to the cardiorenal syndrome. The supratherapeutic INR, elevated bilirubin, and chronic hypotension were assessed to be secondary to alcoholic cirrhosis. Lower extremity computed tomography (CT) with contrast and X-ray of the chest were unremarkable, and ultrasound showed chronic but stable DVT in the mid popliteal and lower femoral veins.

**Table 2:** Pertinent patient-specific labs during hospital admission WBC: white blood cell, RBC: red blood cell, Hgb: hemoglobin, Hct: hematocrit, RDW: RBC distribution width, MCV: mean cell volume, MCH: mean corpuscular hemoglobin, MCHC: mean corpuscular hemoglobin concentration, MPV: mean platelet volume, PT: prothrombin time, INR: international normalized ratio, PTT: partial thromboplastin time, BUN: blood urea nitrogen, AST: aspartate transaminase, ALT: alanine transaminase, TSH: thyroid-stimulating hormone.

Day of admission	1	2	3	4	5	6	7	8
WBC (4.5–11 Thou/cu mm)	11.0	10.6	11.0	8.1	5.9	4.2	4.3	-
RBC (4.6–6.2 Mill/cu mm)	4.34 (L)	4.07 (L)	3.88 (L)	3.81 (L)	3.62 (L)	3.83 (L)	3.95 (L)	-
Hgb (14–18 g/dL)	13.1 (L)	12.1 (L)	11.6 (L)	11.4 (L)	11.0 (L)	11.4 (L)	11.7 (L)	-
Hct (40–54%)	39.4 (L)	37.7 (L)	37.2 (L)	34.5 (L)	31.7 (L)	32.2 (L)	35.5 (L)	-
RDW (11.5–14.5%)	21.3 (H)	21.3 (H)	21.5(H)	20.5 (H)	19.6 (H)	19.9 (H)	20.0 (H)	-
MCV (89–94 fL)	90.8	92.6	95.9	90.6	87.6	89.3	89.9	-
MCH (27–31 pg)	30.2	29.7	29.9	29.9	30.4	29.8	29.6	-
MCHC (32–37 g/dL)	33.2	32.1	31.2 (L)	33.0	34.7	33.3	33.0	-
Platelet (150–450 Thou/cc mm)	175	152	164	148 (L)	122 (L)	135 (L)	103 (L)	-
MPV (8.8–13.5 fL)	12.8	12.0	11.9	12.3	11.8	11.7	11.4	-
Neutro auto (50–65 %)	80.8 (H	79.8 (H)	77.4 (H)	71.3 (H)	72.8 (H)	66.3 (H)	68.3 (H)	-
Lymph auto (30–40%)	13.2 (L)	14.2 (L)	15.5 (L)	21.8 (L)	19.4 (L)	23.1 (L)	24.0 (L)	-
Cardiac enzymes								
Troponin-I (<0.04)	0.03	-	-	-	-	-	-	-
Total CK (30–200 unit(s)/L)	34	-	-	-	-	-	-	-
B-Type natriuretic peptide (<100 pg/mL)	4,624 (H)	-	-	-	-	-	-	-
Coagulation								
PT (9.4–12.5 seconds)	62.0 (H)	48.1 (H)	46.7 (H)	46.2 (H)	38.3 (H)	32.9 (H)	25.3 (H)	-
INR (high > 1)	5.34 (H)	4.13 (H)	4.0 (H)	3.9 (H)	3.28 (H)	2.82 (H)	2.12 (H)	-
PTT (25.1–36.5 seconds)	-	54.4 (H)	-	-	-	-	-	-
Renal function								
Creatinine (0.5–1.2 mg/dL)	1.5 (H)	1.5 (H)	1.3 (H)	1.1	0.9	0.9	0.8	-
BUN (6–20 mg/dL)	49 (H)	48 (H)	47 (H)	39 (H)	30 (H)	26 (H)	20	-
Routine chemistry								
Sodium (136–145 mmol/L)	133 (L)	133 (L)	130 (L)	131 (L)	129 (L)	130 (L)	131 (L)	-
Potassium (3.5–5.1 mmol/L)	4.0	4.2	4.4	3.8	3.0 (L)	3.2 (L)	3.9	-
Chloride (98–107)	95 (L)	97 (L)	97 (L)	94 (L)	93 (L)	95 (L)	96 (L)	-
Glucose (70–110 mg/dL)	103	85	126	78	100	83	73	-
Calcium (8.5–10.5 mg/dL)	8.4 (L)	8.4 (L)	8.0 (L)	7.9 (L)	7.9 (L)	8.0 (L)	7.8 (L)	-
Phosphorus (2.5–4.6 mg/dL)	-	-	-	-	2.5	-	2.3 (L)	-
Albumin level (3.2–5.5 g/dL)	2.2 (L)	2.1 (L)	-	-	-	-	1.7 (L)	-
Total protein (6.7–8.2 g/dL)	7.4	6.7	-	-	-	-	6.4 (L)	-
Alk phosphorus (42–121 units/L)	161 (H)	141 (H)	-	-	-	-	193 (H)	-
Liver function								
AST (5–34 units/L)	22	23	-	-	-	-	47 (H)	-
ALT (10–60 units/L)	25	23	-	-	-	-	7 (L)	-
Bili total (0.2–1.2 mg/dL)	1.3 (H)	1.5 (H)	-	-	-	-	1.0	-
Thyroid study								
TSH (0.34–5.60 mcIU/mL)	9.59 (H)	-	-	-	-	-	-	-
T4 free (0.60–1.60 ng/dL)	-	-	0.63	-	-	-	-	-
Immunology/serology								
C-reactive protein (<0.5 mg/dL)	25.2 (H)	-	-	-	-	-	-	-
Procalcitonin	2.42 (H)	-	-	-	-	-	-	-

The patient was transferred to the intensive care unit and placed on continuous infusions of norepinephrine for hypotension and milrinone for the CHF exacerbation. The patient remained hypotensive and tachycardic throughout the hospital stay. The patient was diagnosed with a lower left extremity DVT in the popliteal and femoral veins. Blood cultures were drawn for cellulitis with possible secondary sepsis before administering intravenous cefalozin. One set of two blood cultures was positive for Staphylococcus epidermidis growth, consistent with collection-associated skin flora. Repeat blood cultures were drawn, and no growth was reported.

The patient decided to leave against medical advice on the seventh day of their hospital stay. Due to the hypotensive state of the patient, a beta-blocker and an angiotensin-converting enzyme inhibitor (ACE-I) were not added at the time of discharge for CHF. The patient was discharged on the same home medications reported prior to hospital admission.

## Discussion

Cardiotoxicity toxicity secondary to methamphetamines and marijuana use

Methamphetamine acts as an indirect agonist on the serotonin, noradrenaline, and dopamine receptors, releasing neurotransmitters into the synapses [8]. The abuse potential for methamphetamine is derived from the initial rush characterized by an elevated or positive mood, increased energy and alertness, and reduced appetite [10]. The most common clinical presentation of methamphetamine toxicity is hypertension, with palpitations, cardiac arrhythmias, and tachycardia [12]. The impact of hypertension and arrhythmias can lead to acute events such as acute coronary syndrome, acute aortic dissection, and sudden cardiac death [8]. Arrhythmias and heart failure are attributed to electrical and structural remodeling of cardiac tissue [9]. Chronic methamphetamine use is linked to the potential for structural and functional changes in myocytes as well as congestive heart failure and cardiomyopathy [7]. Reduced ejection fraction and cardiomyopathy have been significantly observed in methamphetamine use in young adults ages 18 to 51 years old [16]. Although often chronic, rare cases have shown recovery of cardiovascular function with medical treatment and discontinuation of methamphetamine use [12]. The following cardiovascular toxicities have also been reported with methamphetamine use: coronary heart disease, impaired myocardial contractility, acute vasospasm, and atherosclerotic cardiovascular disease [8, 9]. Methamphetamine has been shown to increase the risk of premature and accelerated coronary artery disease (CAD), with underlying cardiovascular disease found in significant proportions of methamphetamine-related deaths [12].

**Figure 2:**
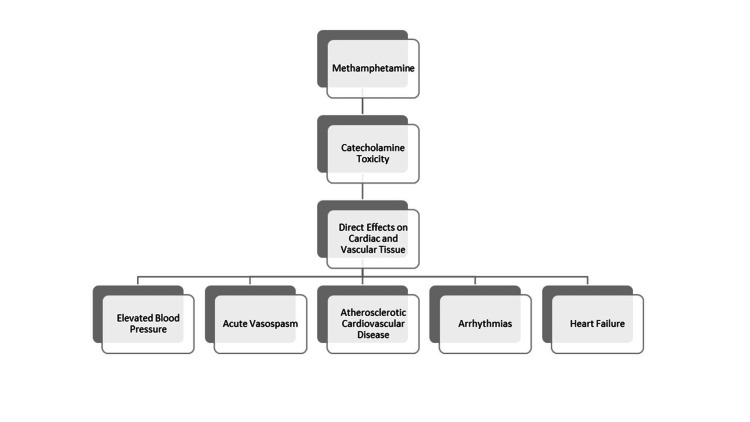
The potential cardiovascular effects of methamphetamine Kevil CG, Goeders NE, Woolard MD, et al. Methamphetamine use and cardiovascular disease: in search of answers. Arterioscler Thromb Vasc Biol. 2019;39(9):1739-46 [10].

As depicted in Figure 2, there are many cardiotoxic effects associated with methamphetamine use. Methamphetamine has been shown to induce prolonged QT changes in ECG [9]. This abnormality can increase the risk of developing ventricular arrhythmias [9]. The structural and cellular changes that methamphetamine causes are associated with cardiac arrhythmias [9]. These structural and cellular changes include fibrosis, inflammation, and electrical remodeling [9].

The endogenous endocannabinoid system (ECS) is comprised of receptors that are responsible for regulating blood pressure and heart rate as well as endocrine and immune responses [17]. The CB1R has been shown to facilitate the development of cardiometabolic disease, while the CB2R has been shown to increase anti-inflammatory effects [18]. Marijuana use has been associated with an increased prevalence of reactive oxygen species, negative inotropic effects, and creating pro-inflammatory endothelial responses [19]. Studies have linked marijuana use to arrhythmia, myocardial infarction (MI), and acute coronary syndrome, among other adverse cardiac complications [17]. Arrhythmias that have been reported to be associated with cannabis use include dose-related sinus tachycardia, sinus bradycardia, atrial fibrillation, and second-degree atrioventricular block [20]. The increased risk of cardiac arrhythmias and MI is potentially due to the increased frequency of marijuana use due to the development of tolerance to THC psychoactive effects. [15] Further evaluation has led to the realization that the short and long-acting metabolites of THC, 11-hydroxy-tetrahydrocannabinol (THC-OH), and 11-carboxy-tetrahydrocannabinol (THC-COOH) alter cell morphology and motility [17]. The proposed mechanism of action of cardiotoxicity secondary to cannabis use is cannabis-induced arteritis, vasospasms, and platelet aggregation [6,15,21]. CB1R and CB2R receptors are also found on platelets and are associated with pro-thrombotic effects [6]. The mechanism of pro-thrombotic effects is thought to be through increased expression of glycoprotein IIb-IIIa and P selectin expression [22]. Cannabis is more commonly associated with coronary thrombus [19,23]. With a relative risk of 2.2, heavy marijuana use is associated with an increased risk of palpitations versus lighter use [6].

**Figure 3:**
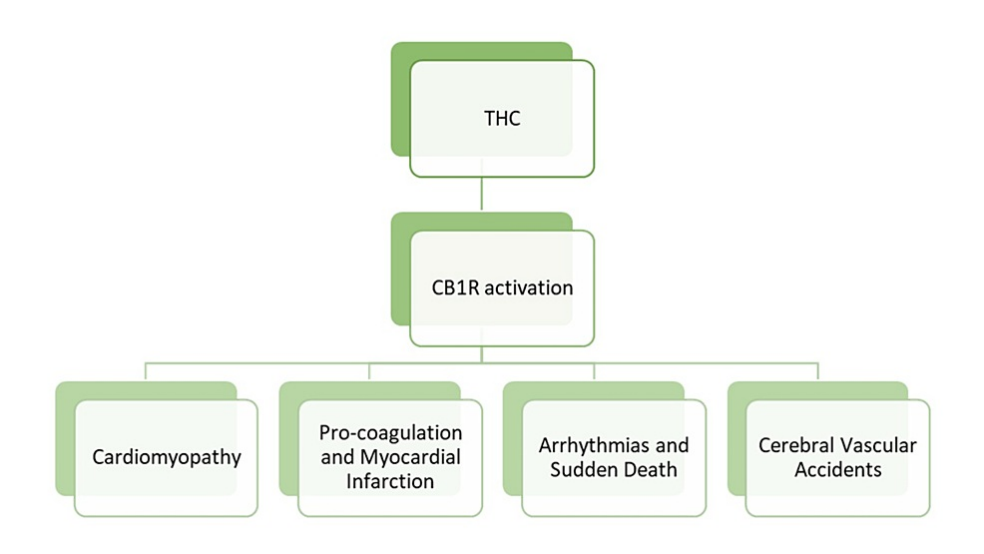
The potential cardiovascular effects of cannabis THC: tetrahydrocannabinol; CB1R: cannabinoid receptor type 1. Page RL, Allen LA, Kloner RA, et al. Medical marijuana, recreational cannabis, and cardiovascular health: a scientific statement from the American Heart Association. Circulation. 2020;142(10):e131-e52.

Marijuana use may lead to various cardiovascular effects, which are summarized in Figure 3. ECG abnormalities reported with marijuana use include premature ventricular contractions, reversible ST-segment changes, and p and T wave changes [6]. In myocardial infarction presentation, ST-segment elevation is a common finding [6]. A decreased amplitude of p waves has also been observed, which is suggestive of atrial abnormality [6]. Arrhythmias associated with cannabis use include ventricular or atrial fibrillation, ectopic ventricular or atrial rhythm, and sinus tachycardia [6].

Toxicology screening for methamphetamines and marijuana

The level of amphetamine metabolite produced in methamphetamine metabolism is substantially lower than the ingested drug concentration [8]. Peak levels of serum amphetamine occur 12 hours after ingestion [8]. From each dose of methamphetamine, 70% is excreted in the urine within 24 hours, and only 10% of that excretion is amphetamine metabolites [8]. The half-life of methamphetamine is approximately 10 hours and it is typically detected in the urine for about 60 hours [8]. No significant difference in the urinary half-life of chronic users versus new methamphetamine exposure has been observed in the literature to date [8]. One case report indicated that urine and blood methamphetamine screening is not adequate due to the short half-life. Therefore, toxicological hair analysis should be added to the panel [24].

A THC dose is excreted by 80-90% within five days of administration [25]. Elimination in urine is about 20%, with the primary metabolite being THC-COOH [25]. The plasma half-life of THC is approximately 4.1 days with chronic use, which is attributed to enterohepatic circulation and slow release from lipid storage, although a single report of a plasma half-life of 12.6 days has been documented during a four-week study [25]. The urinary half-life of cannabis has been reported to be a wide range of three to six days [25]. A higher amount of THC is excreted in feces than in urine [25].

Discussion of case

This case was complex with several high-priority problems, including heart failure exacerbation, lower left extremity swelling and cellulitis, lower left extremity DVT, hemodynamic instability, and electrolyte abnormalities. The presentation of sinus tachycardia was identified on the ECG and the hypotension persisted throughout the hospital stay, requiring vasopressors until the patient left against medical advice. These findings are similar to a case report where a 23-year-old male who also had a history of chronic methamphetamine and marijuana consumption with heart failure presented with the same hemodynamic instability [26]. Sinus tachycardia has been reported in several case reports with either marijuana or methamphetamine exposure, as well as one report with concomitant exposure [20,26]. A limitation of the case study is the possibility that alcohol-induced cardiomyopathy or cirrhotic cardiomyopathy may have also been attributed to the heart failure associated with this patient.

It is plausible that the pro-thrombotic effects of marijuana may have contributed to the hypercoagulable state and history of thrombosis. However, most existing case reports focus on stroke and MI thrombi [19,27]. In addition, the patient's non-compliance with apixaban also may have been a contributor to the DVT present at the time of admission.

One limitation of this case study is the inability to confirm methamphetamine use with a qualitative or quantitative serum or urine toxicology screen. A report on a case of fatal methamphetamine toxicity documented a positive urine methamphetamine screen on admission, but during an autopsy nine days later, methamphetamine was not identified in the urine [24]. They were, however, able to identify methamphetamine in hair toxicological analysis [24]. These findings suggest that hair toxicological analysis could prove to be a more sensitive measure for methamphetamine when determining contributing toxicities [24]. Other contributors to the patient's clinical decline could include homeless status and non-compliance with medications.

It is possible that the cardiorenal syndrome attributed to alcoholic cirrhosis contributed to the observed hemodynamic instability in the case. However, there has been some evidence in the literature that methamphetamine or marijuana use may have the potential to cause hepatotoxicity, which could have contributed to the patient’s hepatic dysfunction. Structural modifications and liver toxicity with signs of hepatic sclerosis have been associated with chronic cannabis use [17]. The mechanism of cannabis hepatotoxicity has been postulated to be through action on CB1R found in hepatic cells [21]. Methamphetamine is considered a hepatotoxic synthetic amine [28]. The proposed mechanism of methamphetamine hepatotoxic action is through increased neurotransmitter efflux, generation of reactive metabolites, oxidation of biogenic amines, mitochondrial impairment, apoptosis, and hyperthermia [28,29].

## Conclusions

Urine drug screen protocols to assess for methamphetamine toxicity that includes only urine and serum assessment are insensitive to recent methamphetamine exposure. Hair toxicological testing should be an option considered when methamphetamine use is suspected, allowing for a more sensitive analysis of use.

Very few published references specifically look at the effects of concomitant use of methamphetamine and cannabis on cardiovascular health. While rare cases have seen improved cardiovascular function with the discontinuation of methamphetamine, cardiomyopathy is often chronic. The reversible nature of the cardiotoxic effects of concomitant marijuana and methamphetamine use has not been documented. Analysis of this case demonstrated that the presentation of cardiotoxicity may persist even after discontinuation of the drugs. Most available sources were from several years ago and were extremely limited in both scope and external validity. The need for further study in this area of medicine is evident in the lack of reliable, applicable primary studies.
